# Review of Cognitive Characteristics of Autism Spectrum Disorder Using Performance on Six Subtests on Four Versions of the Wechsler Intelligence Scale for Children

**DOI:** 10.1007/s10803-021-04932-x

**Published:** 2021-03-07

**Authors:** Mizuho Takayanagi, Yoko Kawasaki, Mieko Shinomiya, Hoshino Hiroshi, Satoshi Okada, Tamiko Ino, Kazuko Sakai, Kimiko Murakami, Rie Ishida, Kaoru Mizuno, Shin-Ichi Niwa

**Affiliations:** 1 Department of Child and Adolescent Psychiatry, Arisawabashi Hospital, 5 Fuchu-machi Haneshin, Toyama, Toyama 9392704 Japan; 2 Musashino Child Development Clinic, Tokyo, Japan; 3 Fukushima Medical University, Fukushima, Japan; 4 Hokkaido University, Hokkaido, Japan

**Keywords:** Autism spectrum disorder (ASD), Cognitive function, Intelligence ability, Systematic review, Wechsler scales

## Abstract

This study was a systematic review of research using the Wechsler Intelligence Scale for Children (WISC) with Autism Spectrum Disorder (ASD) to examine cognitive characteristics of children with ASD beyond the impact of revisions based on WISC and diagnostic criteria changes. The classic “islets of ability” was found in individuals with full-scale IQs < 100. The “right-descending profiles” were observed among high IQ score individuals. High levels on the Block Design and low Coding levels were consistently found regardless of the variation in intellectual functioning or diagnosis. This review identified patterns of cognitive characteristics in ASD individuals using empirical data that researchers may have previously been aware of, based on their experiences, owing to the increased prevalence of ASD.

## Introduction

In 1943, autism spectrum disorder (ASD) was first described as an “autistic disorder of affective contact” (Kanner 1943). Since then, many similar cases have been reported worldwide, with this rapid increase in its prevalence in recent years attracting considerable attention (Fombonne 2005). The prevalence of ASD was initially approximately 4.5 or 10–20 persons in a population of 10,000 (Lotter 1966; Wing et al. 1976). However, over the past two decades, it has steadily increased, from 67 to 131–293 persons per 10,000 in the United States (Baio et al. 2018; Bertrand et al. 2001), 48–161 in Japan (Honda et al. 2005), 116 in the UK (Baird et al. 2006), and 264 persons per 10,000 in South Korea (Kim et al. 2011). This was primarily due to an increase in high IQ score cases without intellectual disabilities (Charman et al. 2011; Fombonne 2009).

During this period, the diagnostic criteria for ASD have changed. They were initially based on a report by Kanner (1943), followed by wide use of Rutter’s criteria internationally (Rutter 1978). In 1980, the third edition of the Diagnostic and Statistical Manual of Mental Disorders (DSM-III; American Psychiatric Association 1980) categorized ASD as a subtype of “infantile autism” under pervasive developmental disorders (PDD). Thereafter, in the DSM-IIIR (American Psychiatric Association 1987), the diagnostic label for the subtype was changed to “autistic disorder,” and pervasive developmental disorder, not otherwise specified (PDDNOS) was added. Subsequently, in the DSM-IV (American Psychiatric Association 1994), “Asperger’s disorder” was introduced as individuals having no clinically significant delays in language development. The DSM-5 (American Psychiatric Association 2013) adopted the term ASD while the diagnostic terms “Asperger’s disorder” and “PDD” were removed.

The International Classification of Disease and Related Health Conditions (ICD) is another internationally recognized diagnostic guideline that has used the diagnostic term PDD with subtypes “childhood autism” and “Asperger’s syndrome” in ICD-10 (ICD-10; World Health Organization [WHO] 1992) since the 1990s. However, in an attempt at harmonization, the ICD-11(World Health Organization [WHO] 2018) adopted a similar disease classification as the DSM-5, including the term ASD. Although ASD’s diagnostic criteria and disease classification changed across the different editions of DSM and ICD, they presently have identical criteria.

The Wechsler scales are the most widely used measures of intelligence and have been translated, adapted, and standardized in many countries worldwide. The Wechsler Adult Intelligence Scale (WAIS; Wechsler 1939) was first developed after the Wechsler Intelligence Scales for Children (WISC; Wechsler 1949). Later, the WISC was revised several times, including the WISC-R (Wechsler 1974), WISC-III (Wechsler 1991), WISC-IV (Wechsler 2003), and WISC-V (Wechsler 2014). These revisions have encompassed various changes, eliminations, and incorporations of new tests even among the subtests. In the first edition of WISC, two index scores, verbal IQ (intelligence quotient), and Performance IQ, were calculated to identify intra-individual differences, along with Full-Scale IQ scores. As a result of the repeated revision of WISC, a procedure for analyzing intra-individual differences was created with four indexes: VCI (Verbal Comprehension Index), POI (Perceptual Organization Index), FDI (Freedom from Distractibility Index), and PSI (Processing Speed Index) (Wechsler 2003). Recently, the following five indexes were created based upon the Cattell-Horn-Carroll theory in WISC-V: VCI, VSI (Visual Spatial Index), FRI (Fluid Reasoning Index), WMI (Working Memory Index), and PSI (Wechsler 2014). To interpret results, both normative and personal strengths and weaknesses among the indexes were identified. Interpretation of fluctuations in the child’s index profile offers reliable and meaningful information regarding WISC performance because it identifies strong and weak areas of cognitive functioning relative to both same-age peers from the normal population (inter-individual approach) and the child’s own overall ability level (intra-individual approach). The WISC results provide clinically meaningful information in areas to develop individual support plans and treatment programs for children with neurodevelopmental disorders (Flanagan and Kaufman 2009).

The oldest reports on the WISC and ASD were by Gillies (1965) and Wassing (1965), both of which revealed low verbal-ability test scores in ASD cases. Later, Lockyer and Rutter (1970) reported the results of their study spanning 5–15 years of follow-ups and including 63 pediatric psychiatric cases that were diagnosed during the 1950s in the UK. Based on the WISC and WAIS subtest profiles from these cases, Lockyer and Rutter identified cognitive profiles common to ASD that Comprehension levels are low, while Block Design, Object Assembly, and Digit Span levels are high. Among them, Block Design has peaks. This pattern was decidedly noticeable among children with pronounced language delay. Lockyer and Rutter (1970) referred to this pattern of characteristics as the “islets of ability.” Thereafter, high Block Design scores and low Comprehension scores have consistently been reported and widely recognized as a typical cognitive profile of ASD (Asarnow et al. 1987; Ehlers et al. 1997; Freeman et al. 1985; Ghaziuddin and Mountain-Kimchi 2004; Happe 1994; Koyama et al. 2006, 2009; Mayes and Calhoun 2003; Shah and Firth (1993); Siegel et al. 1996; Szatmari et al. 1990).

Various hypotheses have been put forward regarding the relationship between ASD’s WISC profile and cognitive impairment. Lockyer and Rutter (1970) interpreted that high performance on the Block Design observed in the islets of ability were due to good perceptual organization in ASD. Based on the low verbal scores, they also hypothesized that ASD was caused by a central disorder of language and perception of sounds (Lockyer and Rutter 1970). Shah and Frith (1993) argued that autistic children do well on only those tasks that favor a piecemeal processing style, where children with no disorders are impeded by their tendency to look for overall meaning or be captured by the global or gestalt form at the expense of the local parts or details, and pointed out that weak central coherence was observed among children with autism (Shah and Frith 1993). Happe (1994) considered that the peak of Block Design in islets of ability was not due to only good perceptual organization but also due to the manifestation of their relatively local, as opposed to global, processing style by their weak central coherence (Happe 1994).

While Lockyer and Rutter (1970) focused on the islet of ability, they did not discuss the WISC profile with respect to Coding. In fact, the lowest subtest score in their data was Coding (Lockyer and Rutter 1970). For Coding, the child works within a specific time limit and uses a key to copy symbols that correspond with simple geometric shapes or numbers. In addition to processing speed, the subtest measures visual perception, visuomotor coordination, cognitive flexibility (shifting rapidly from one pair to another), attention skills, and possibly, motivation (Sattler 2004; Weiss et al. 2015). Factors such as problems of motor coordination (Mayes and Calhoun 2003) and cognitive flexibility (Hedvall et al. 2013) were pointed out for low Coding and PSI values in children with ASD.

Initially, Lockyer and Rutter (1970) saw the islets of ability in the WISC profile of ASD, which was associated with intellectual disability and demonstrated particularly low verbal-ability test scores. Later, Happe (1994) and Shah and Frith (1983, 1993) proposed good perceptual organization and weak central coherence, which were considered to be consistent with ASD’s WISC profile. More recently, cognitive characteristics, such as cognitive inflexibility and impairment of mentalizing (Baron-Cohen et al. 1985; Baron-Cohen 2004), were also proposed, and it has been considered that these, along with the WISC profile among children with ASD are consistent. However, within the half-century since the concept of ASD was proposed, the proportion of high IQ scores displayed by children with ASD increased (Fombonne 2009). Consequently, it has been observed that recent WISC profiles of ASD have changed from what had historically been identified as the WISC profile for children with ASD (Siegel et al. 1996; Mayes and Calhoun 2008; Charman et al. 2011). However, there are no reports that systematically discuss and examine this point.

WISC is a test with reliability and validity confirmed by its prolonged widespread use (Wechsler 1974, 2003), and the Composite Scale has been stable over time (Bartoi et al. 2015; Kieng et al. 2017). The content validity and constructs validity of the FSIQ and Composite Scale have been repeatedly verified, and evidence of interpretation methods have been accumulated. Thus, analyses of indexes with reliability and validity have been recommended for the utility of intra-individual analysis in recent years. Meanwhile, an intra-individual analysis using subtests is not recommended, due to low reliability and lack of evidence of interpretation validity of such an approach (Flanagan and Kaufmann 2009). However, the cognitive characteristics of ASD, such as weak central coherence and cognitive flexibility, which have been pointed out, cannot be captured by observing the current Composite Scales —VCI (Verbal Comprehension Index), POI (Perceptual Organization Index), FDI (Freedom From Distractibility Index), PSI (Processing Speed Index) (Wechsler 2003), VSI (Visual Spatial Index), FRI (Fluid Reasoning Index), WMI (Working Memory Index), and PSI (Wechsler 2014). It is difficult to derive an answer by discussing only the Composite Scale in response to the question of whether the cognitive characteristics of ASD that were identified on the subtest profile in previous WISC versions, including the islets of ability, are also displayed by current children with ASD. In order to supplement the instability of subtest, it is crucial to extract subtests common to the studies reported to date, find common features from multiple studies, and examine them. Therefore, in this study, we conducted a systematic literature search for empirical studies on WISC in ASD. To eliminate the effects of the revision of WISC from the extracted papers, six subtests—commonly employed by WISC, WISC-R, WISC-III, and WISC-IV (i.e., Similarities, Vocabulary, Comprehension, Block Design, Digit Span, and Coding)—were extracted, and the mean scores were compared. The purpose of this study is to delineate the cognitive characteristics of ASD beyond the impact of changes in diagnostic criteria and the revisions of the WISC. We hope that this review will provide updated information regarding recent WISC profiles of ASD to current clinicians engaged in child psychiatry.

## Methods

### Literature Review

This review selected literature adopting the preferred reporting items for systematic reviews and meta-analyses (PRISMA) statement (Liberati et al. 2009). Three databases of PubMed, PsychINFO, and Ichu-Shi were used for literature identification, and the searches were conducted between December 19–20, 2018. The selection criteria comprised all literature published before December 20, 2018, including peer-reviewed journal articles written in English and papers with one or more of the following keywords: autism, pervasive developmental disorder, PDD, Asperger, WISC, or Wechsler Intelligence Scale for Children (PubMed, *n* = 569; PsychINFO, *n* = 3,817; and Ichu-Shi, *n* = 37). After excluding duplicates, 4,208 papers were initially identified and from among those, 51 papers that discussed the mean scores of the subtests of WISC, WISC-R, WISC-III, or WISC-IV for participants with ASD or PDD were selected. From these, studies that did not include all the mean scores of six subtests of interest, studies using WAIS, and a study evaluating only PDD-NOS participants were excluded. Thus, we selected 27 papers.

### Comparison of Scores

We compared the mean scores of the commonly employed six subtests (i.e., Similarities, Vocabulary, Comprehension, Block Design, Digit Span, and Coding). Moreover, the subtest profiles were compared between different intellectual levels and diagnoses.

## Results

### Systematic Review to Extract Relevant Studies

The 51 studies were extracted, following the PRISMA statement for systematic review. Table 1 presents the names of authors, year of publication, participants groups, the ultimately analyzed participants, diagnoses within the paper, number of cases, the mean age and age range of the target group, mean FSIQ, and the tests conducted. The ultimately analyzed participants refer to the group that met all selection criteria (to be further discussed later), and thus, were included in the final analysis. These 51 studies were published between 1970 and 2017, and the total number of participants ranged from 9 (Bartak et al. 1975) to 166 (Mouga et al. 2016). There were 4 WISC studies, 17 WISC-R studies, 23 WISC- III studies, and 10 WISC- IV studies. The Participant Groups refer to the groups that the studies include, multiple participant groups, which were separated into categories by diagnoses, test versions, and intellectual levels. For studies in which the same authors had assessed the same participants, only one was adopted. Finally,14 studies including mixed WAIS results were excluded, and another nine studies were removed because they did not include all the mean scores of six subtests of interest. Readers are encouraged to refer to the detailed reasons for exclusion that are stated below in Table 1. The studies by Bölte and Poustka (2004), Koyama et al. (2009), Kumazaki et al. (2015), and Calero et al. (2015) included results obtained after categorizing participants into groups by either gender or treatment response. However, these results were treated as one group.

**Table 1:** Studies on the WISC related to autism spectrum extracted by systematic review

Reference Number	Authors and Year of Publication	Participant Groups	Ultimately Analyzed Participants	Diagnosis	*N*	Age (y) *M*	Age (y) Range	FSIQ *M*	Test(s)
1	Lockyer and Rutter (1970)			Infantile psychosis	21	15.7	–	74.2	WISC/WAIS
2	Bartak et al. (1975)		†	Infantile autism	9	–	4.5–9.9	–	WISC
3	Tymchuk et al. (1977)			Childhood psychosis	20	15.9	–	88	WISC/WAIS
4	Freeman et al. (1985)		†	Autistic children	21	8.8	6–12	97.4	WISC-R
5	Ohta (1987)		†	Infantile autism	16	10.2	6–14	72.1	WISC
6	Asarnow et al. (1987)			Infantile autism	23	10.4	–	91.4	WISC-R
7	Lincoln et al. (1988)		†	Infantile autism	13	–	8–12	68.5	WISC-R
8	Szatmari et al. (1990)	S1		Autism	17	22.8	7–32	82.2	WISC-R/WAIS-R
		S2		Asperger’s syndrome	26	14.3	8–18	86.6	WISC-R/WAIS-R
9	Allen et al. (1991)		†	Autistic children	20	10.3	6–12	68.4	WISC-R
10	Venter et al. (1992)			Autism	58	14.7	10–37	79.2	WISC-R/WAIS-R
11	Happe (1994)			Autism	51	15.4	7–25	62.1	WISC-R/WAIS
12	Siegel et al. (1996)		†	Autism	45	10.1	6–16	96	WISC-R
13	Ehlers et al. (1997)	E1	†	Infantile autism/ Autistic disorder	40	9.9	6.1–15.8	78.8	WISC-R
		E2	†	Asperger syndrome	40	9.8	5.3–15	102.5	WISC-R
14	Manjiviona and Prior (1999)	M1	†	Autism	21	11.6	7–15	88.61	WISC-R
		M2		Asperger syndrome	35	10.4	6–17	102.6	WISC-R/WAIS-R
15	Ozonoff et al. (2000)	O1		Autism	23	13.3	6.6–20.9	108.9	WISC-III/WAIS-III
		O2		Asperger syndrome	12	13.9	6.6–20.9	115.6	WISC-III/WAIS-III
16	Nydēn et al. (2001)		†	Asperger syndrome	13	9.8	6.6–11	106.8	WISC-III
17	Bōlte et al. (2002)			Autism	20	16.8	14–21.3	82.5	WISC-R/WAIS-R
18	Mayes and Calhoun (2003)			Autistic disorder	53	8.5	6–15	88.4	WISC-III
19	Bölte and Poustka (2004)			Autism	59	17.9	6.4–49.4	78.1	WISC-R/WAIS-R
20	Cederlund and Gillberg (2004)			Asperger syndrome	98	11.4	5.6–24.6	101	WISC-R/WSIC-III/ WAIS-R
21	Ghaziuddin and Mountain-Kimchi (2004)	G1		Autism	12	12.4	–	92.2	WISC-III/WAIS-R
		G2		Asperger syndrome	22	12.2	–	103.3	WISC-III/WAIS-R
22	Mayes and Calhoun (2004)		†	Autism	93	9	6–16	103	WISC-III
23	de Bruin et al. (2006)	B1	†	Autism	13	8.6	6–12	88.9	WISC-R
		B2	†	Asperger syndrome	11	8.6	6–12	106.3	WISC-R
24	Koyama et al. (2006)			PDDNOS	27	8	5.6–13.8	94.9	WISC-III
25	Williams et al. (2006b)		†	Autism	38	11.7	8–16	103.8	WISC-III
26	Williams et al. (2006a)			Autism	56	11.4	8–15	104.1	WISC-III
27	Koyama et al. (2007)	K1		Autistic children	37	12.6	5.4–30.3	94.6	WISC-R/WISC-III/WAIS-R
		K2		Asperger syndrome	36	12.8	5.6–30.5	98.3	WISC-R/WISC-III/WAIS-R
28	Muraru et al. (2007)		†	PDD	53	9.2	–	97.8	WISC-III
29	Zhang et al. (2007)		†	PDD	135	9	5–16	92.9	WISC-III
30	Koyama and Kurita (2008)		†	Asperger syndrome	28	9.3	5–13	102.1	WISC-III
31	Mayes and Calhoun (2008)		†	Autism	54	8.2	6–14	101	WISC-IV
32	Koyama et al. (2009)		†	PDD	142	8.9	–	96.3	WISC-III
33	Inada and Kamio (2010)			ASD	48	12.5	–	93.4	WISC-III
34	Noterdaeme et al. (2010)	N1		Autism	55	10.6	6.1–19.5	94	WISC-III/WAIS
		N2		Asperger’s syndrome	57	11.2	6.8–19.9	104.1	WISC-III/WAIS
35	Charman et al. (2011)			ASD	127	11.5	9.8–14.5	75.5	WISC-III
36	Foley-Nicpon et al. (2012)	F1	†	Autism	18	–	6–16.2	120.3	WISC-IV
		F2	†	Asperger syndrome	21	–	6–16.2	124.9	WISC-IV
37	Merchan-Naranjo et al. (2012)			Asperger syndrome	29	13	7–17	96.9	WISC-R/WAIS-III
38	Oliveras-Rentas et al. (2012)		†	ASD	56	9.1	6–15	97.6	WISC-IV
39	Planche and Lemonnier (2012)	P1		Autism	15	8.1	6–10.1	98.1	WISC-III
		P2		Asperger’s syndrome	15	8	6–9.8	105.5	WISC-III
40	McGonigle-Chalmers and McSweeney (2013)			ASD	15	13.7	12.5–15.2	–	WISC-III
41	Reinvall et al. (2013)			Asperger syndrome	20	13.5	12–16.1	103.2	WISC-III
42	Kuriakose (2014)		†	ASD	23	11.2	7.1–15.6	80	WISC-IV
43	Matsuura et al. (2014)		†	ASD	11	12	–	105.4	WISC-IV
44	Zielińska et al. (2014)			Autism	35	9.4	–	97.1	WISC-R
45	Calero et al. (2015)		†	Asperger syndrome	45	9.6	7–13	102.3	WISC-IV
46	Kumazaki et al. (2015)		†	ASD	46	7.5	5–9	97.6	WISC-III
47	Nader et al. (2015)	N1	†	Autism	51	10.5	6–16	90.6	WISC-III
		N2	†	Asperger syndrome	15	11.5	7–15	99.4	WISC-III
		N3	†	Autism	51	10.6	7–15	90.7	WISC-IV
		N4	†	Asperger syndrome	15	10.6	7–15	98.3	WISC-IV
48	Mouga et al. (2016)	M1	†	ASD	58	9.8^§^	6–16.9	60.9	WISC-III
		M2	†	ASD	166	9.8^§^	6–16.9	96.5	WISC-III
49	Li et al. (2017b)		†	ASD	32	10.3	6–16	94.4	WISC-IV
50	Li et al. (2017a)			ASD	31	10.2	6–16	94.7	WISC-IV
51	Stack et al. (2017)		†	ASD	134	–	6–15.7	97.8	WISC-IV

*Participant Groups:* When a paper by one author contains multiple participant groups, the group names are distinguished by giving the initial letter of the author name of the group and numbers. E.g.) For Szatmari, there are 2 groups with different diagnostic groups, which are S1 and S2. For Nader, there are 4 groups with different diagnostic groups and WISC test versions, which are N1, N2, N3, and N4

The “Ultimately Analyzed Participants” refers to the group that met all selection criteria and became this study’s ultimate analysis participants. † indicates the selected groups

Reasons for exclusion) Paper numbers 1,3,8,10,15,17,19,20,21,27,34,37 and M2 of paper number14 were excluded because they included WAIS. Paper number 6 was excluded because of the original study excluded the numerical values, while the mean scores were provided elsewhere (Happe 1994). Paper number 18 was excluded because the subtest results were standardized, and paper number 22 included past examples of their research. Paper number 24 was excluded because only participants with PDD-NOS were included in the study. Paper number 26 was excluded because of lack of similarities and Comprehension scores. Paper numbers 33,35,39,44 were excluded because of lack of Digit Span scores. Paper number 40 was excluded because the study did not include any verbal subtests. Paper number 41 was excluded because of lack of Vocabulary and Digit Span scores. Since paper numbers 49 and 50 seemed to examine the same participants, only paper number 49 was used and paper number 50 was excluded

*M* mean, *ASD* Autism Spectrum Disorder, *FSIQ* Full Scale Intelligence Quotient, *PDD* Pervasive Developmental Disorder, *PDDNOS* Pervasive Developmental Disorder Not Otherwise Specified, *WAIS* Wechsler Adult Intelligence Scale, *WISC* Wechsler Intelligence Scale for Children

^§^The mean Age in Mouga was calculated by summing all cases

Overall, 27 papers were ultimately selected. Of these, those that included multiple participants were separated into categories by diagnosis and intellectual level, and each category was counted as a separate group. Finally, the review participants comprised a total of thirty-four groups that were a part of the “ultimately analyzed participants (†).”

### Mean Subtest Scores, Diagnostic Criteria, and Diagnoses

Table 2 provides details for the 34 studies that comprised the ultimately analyzed participants. Of these, the WISC reported in Bartak et al. (1975) and Ohta (1987), as well as the WISC-R in Lincoln et al. (1988), moreover, Allen et al. (1991), showed low Similarities, Vocabulary, and Comprehension scores, combined with particularly low Comprehension scores, which created a notable trough. Block Design displayed high scores that created a peak, while Digit Span and Coding indicated comparatively low scores. Thus, these groups demonstrated classic islets of ability profiles. While some of the remaining 30 groups displayed troughs in Comprehension scores and peaks in Block Design, the verbal levels did not stand out as consistently low. In fact, some groups demonstrated high verbal levels, suggesting different patterns.

**Table 2:** Mean scores of six subtests, diagnostic criteria, and diagnosis of the ultimate group analyzed

Author(s) and Year of Publication	Test	*N*	FSIQ	Similarities	Vocabulary	Comprehension	Block Design	Digit Span	Coding	Diagnostic Criteria	Diagnosis
Bartak et al. (1975)	WISC	9		3.8	4.1	2.7^‡^	12.4^†^	7.2	7.7	Rutter (1971)	Infantile autism
Ohta (1987)	WISC	16	72.1	4.4	4.4	1.4^‡^	10.6^†^	6.1	6.6	DSM-III	Infantile autism
Freeman et al. (1985)	WISC-R	21	97.4	10.4	8.1	6.5^‡^	12.1^†^	8.8	7.7	DSM-III	Autistic children
Lincoln et al. (1988)	WISC-R	13	68.5	4.2	1.9	1.4^‡^	10.2^†^	5.2	4.8	DSM-III	Infantile autism
Allen et al. (1991)	WISC-R	20	68.4	3.7	2.3	1.3^‡^	11.2^†^	5.0	4.4	DSM-III-R	Autistic children
Siegel et al. (1996)	WISC-R	45	96.0	10.6	9.0	7.5^‡^	11.3^†^	9.6	7.5	DSM-III-R	Autism
Ehlers et al. (1997) Participant E1	WISC-R	40	78.8	8.2	6.8	6.3	9.1^†^	7.8	5.5^‡^	DSM-III/ DSM-III-R	Infantile autism/ Autistic disorder
Ehlers et al. (1997) Participant E2	WISC-R	40	102.5	12.3^†^	12.0	11.2	10.5	10.4	7.8^‡^	Gillberg and Gillberg (1989)	Asperger syndrome
Manjiviona and Prior (1999)	WISC-R	21	88.6	9.9	7.6	7.2	10.2^†^	7.4	6.0^‡^	DSM-III-R/ ICD-10	Autism
de Bruin et al. (2006) Participant B1	WISC-R	13	88.9	8.8^†^	7.7	7.9	7.8	8.4	5.8^‡^	DSM-IV	Autism
de Bruin et al. (2006) Participant B2	WISC-R	11	106.3	12.5^†^	11.1	9.1	10.5	9.5	6.6^‡^	DSM-IV	Asperger syndrome
Nydēn et al. (2001)	WISC-III	13	106.8	11.9	15.1^†^	11.8^‡^	12	8.1	8.2	DSM-IV^§^/ Gillberg (1991)	Asperger syndrome
Mayes and Calhoun (2004)	WISC-III	93	103.0	12.2^†^	11.4	9	11.6	8.9	7.5^‡^	DSM-IV	Autism
Williams et al. (2006b)	WISC-III	38	103.8	12.1^†^	11.3	7.5^‡^	11.7	10.4	8.4	No description^§^	Autism
Muraru et al. (2007)	WISC-III	53	97.8	9.4	9.1	9.2	10.4^†^	9.1	8.2^‡^	DSM-IV	PDD
Zhang et al. (2007)	WISC-III	135	92.9	9.3	8.8	8.4	9.4^†^	8.9	7.7^‡^	DSM-IV	PDD
Koyama and Kurita (2008)	WISC-III	28	102.1	10.7	10.5	9.1	11.9	12.4^†^	8.5^‡^	DSM-IV	Asperger syndrome
Koyama et al. (2009)	WISC-III	142	96.3	9.9	9.0	7.9^‡^	11.2^†^	11.2	9.0	DSM-IV/ ICD-10	PDD
Nader et al. (2015) Participant N1	WISC-III	51	90.6	9.6	7.2	5.2^‡^	12.2^†^	8.0	6.8	DSM-IV	Autism
Nader et al. (2015) Participant N2	WISC-III	15	99.4	11.9	13.3^†^	7.6	10.7	12.7	6.4^‡^	DSM-IV	Asperger syndrome
Kumazaki et al. (2015)	WISC-III	46	97.6	9.5	9.3	8.7^‡^	11^†^	10.3	9.1	DSM-IV/ ICD-10	ASD
Mouga et al. (2016) Participant M1	WISC-III	58	60.9	5.9	3.7	3.1^‡^	6.3^†^	5.7	3.9	DSM-5	ASD
Mayes and Calhoun (2008)	WISC-IV	54	101.0	12.9^†^	11.5	9.3	11.6	8.4	6.6^‡^	DSM-IV	Autism
Foley-Nicpon et al. (2012) Participant F1	WISC-IV	18	120.3	13.6^†^	13.3	11.4	12.8	11.9	9.5^‡^	DSM-IV-TR	Autism
Foley-Nicpon et al. (2012) Participant F2	WISC-IV	21	124.9	15.2	15.6^†^	12.9	13.6	12.6	7.4^‡^	DSM-IV-TR	Asperger syndrome
Oliveras-Rentas et al. (2012)	WISC-IV	56	97.6	11.8^†^	10.3	7.9	10.5	9.5	7.3^‡^	DSM-IV	ASD
Kuriakose (2014)	WISC-IV	23	80.0	8.4	7.0	4.9^‡^	9.5^†^	7.4	5.7	DSM-IV-TR	ASD
Matsuura et al. (2014)	WISC-IV	11	105.4	11.7	10.4	9.8	12.2^†^	11.9	9.2^‡^	DSM-IV-TR	ASD
Nader et al. (2015) Participant N3	WISC-IV	51	90.7	8.4	8.2	5.8^‡^	11^†^	7.6	7.7	DSM-IV	Autism
Nader et al. (2015) Participant N4	WISC-IV	15	98.3	12.5	13.3^†^	9.3	9.5	8.3	6.7^‡^	DSM-IV	Asperger syndrome
Calero et al. (2015)	WISC-IV	45	102.3	13.2^†^	12.8	10.1	11.3	10.9	7.8^‡^	No description^§^	Asperger syndrome
Li et al. (2017b)	WISC-IV	32	94.4	10.7	8.1	8	12.3^†^	8.9	7.7^‡^	DSM-5	ASD
Stack et al. (2017)	WISC-IV	134	97.8	11.9^†^	10.3	8.7	10.4	9.3	7.6^‡^	DSM-IV-TR/ ICD-10	ASD

*ASD* Autism Spectrum Disorder, *DSM* Diagnostic and Statistical Manual of Mental Disorders, *FSIQ* Full Scale Intelligence Quotient, *ICD* International Classification of Disease and Related Health Conditions, *PDD* Pervasive Developmental Disorder, *WISC* Wechsler Intelligence Scale for Children

^†^Added to the highest score of the six subtests. ^‡^ Added to the lowest score of the six subtests

^§^This case included in Nydēn used “Asperger’s Syndrome” as its diagnostic name, it was further explained that ‟they also met DSM-IV criteria for Asperger’s Syndrome with the exception that language development and curiosity about the environment was not normal in all cases.” Williams and Calero did not describe the diagnostic criteria employed

### Comparison of Three Groups

#### Comparison of Six Subtest Profiles by Intellectual Level (Fig. 1)

**Fig. 1:**
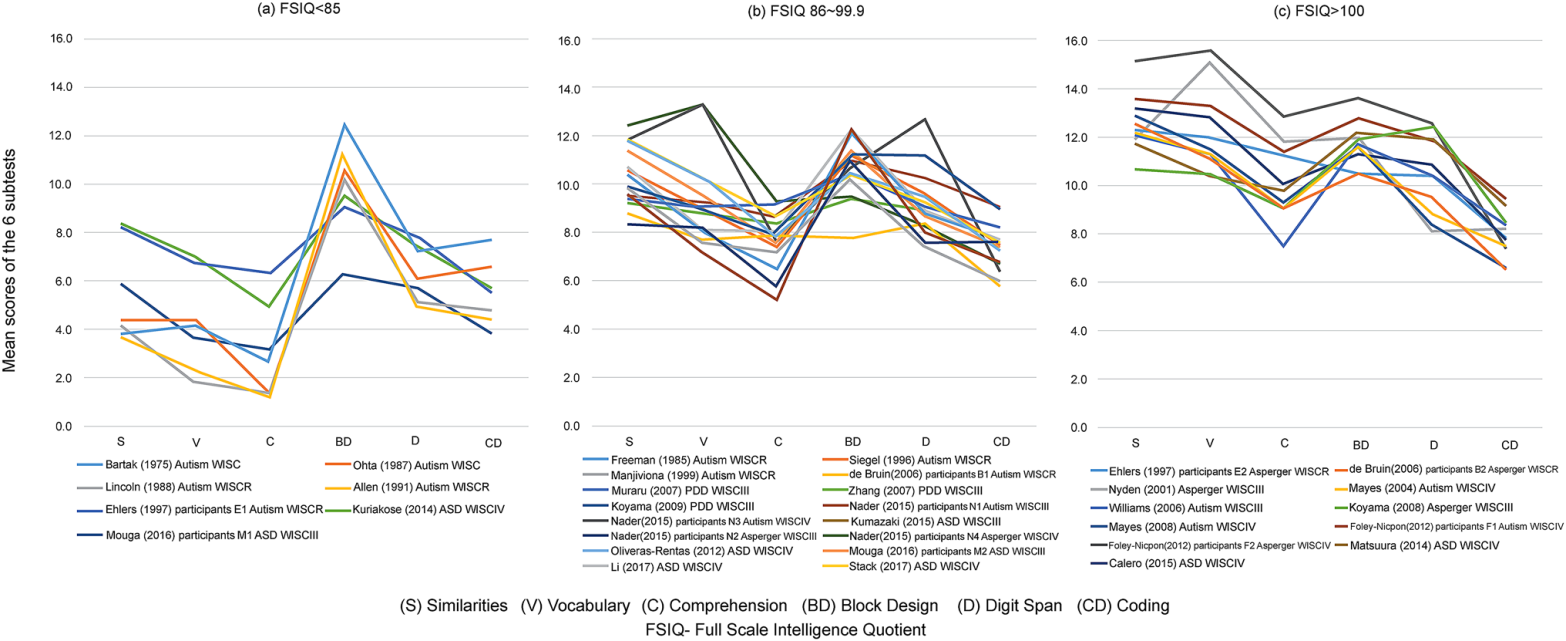
Comparison of six subtest profiles of the three groups based on intellectual level. **a** Seven groups with a mean FSIQ of ≤ 85. **b** Sixteen groups with a mean FSIQ of ≥ 86 and < 100. **c** Eleven groups with a mean FSIQ of ≥ 100. The order of subtests in figures for this study followed the order in the present WISC-IV manual. ASD, Autism Spectrum Disorder; PDD, Pervasive Developmental Disorder; WISC, Wechsler Intelligence Scales for Children

Based on the mean FSIQ of the ultimately analyzed participants, the groups were divided into three ranges: (a) mean FSIQ of ≤ 85; (b) mean FSIQ of 86–100, and (c) mean FSIQ of > 100. The relationships between intellectual levels and the six subtest profiles were then examined. The three graphs in Fig. 1 indicate the six subtest profiles. Among the seven groups with a mean FSIQ of ≤ 85, Allen et al. (1991), Bartak et al. (1975), Lincoln et al. (1988), and Ohta (1987) exhibited classic islets of ability profiles. Although in the report by Ehlers et al. (1997) participants with autism diagnoses (E1), and in that by Kuriakose (2014) and Mouga et al. (2016) participants with low IQ scores (M1) did not demonstrate typical profiles; there were troughs in Comprehension and peaks in Block Design. Therefore, overall, the groups exhibited classic islets of ability profiles. For the sixteen groups with mean FSIQ scores between 86–100, the included profiles were diverse though there were many classic islets of ability profiles with Comprehension troughs and Block Design peaks. Among the 11 groups with a mean FSIQ of ≥ 100, high verbal scores on Similarities and Vocabulary stood out. Apart from Nydēn et al. (2001) and Williams et al. (2006b), Coding had the lowest scores in the other nine groups, displaying right-descending profiles.

#### Comparison by Diagnosis and Intellectual Level (Fig. 2)

For the three intellectual-level–based groups, further examination was conducted by subdividing them by diagnosis into three groups: autism diagnoses (including autism, autistic children, and infantile autism), PDD or ASD diagnoses, and Asperger’s diagnoses. Among the groups with a mean FSIQ of ≤ 85—excluding Kuriakose (2014) and Mouga et al. (2016) participants with low IQ scores (M1) with ASD diagnoses—all represented autism diagnoses with no cases corresponding to Asperger’s disorder. Although low verbal levels were not detected by Ehlers et al. (1997) participants with autism diagnoses (E1), a trough-and-peak trend of low Comprehension levels and high Block Design levels was found, thereby confirming a typical islets of ability profile.

**Fig. 2:**
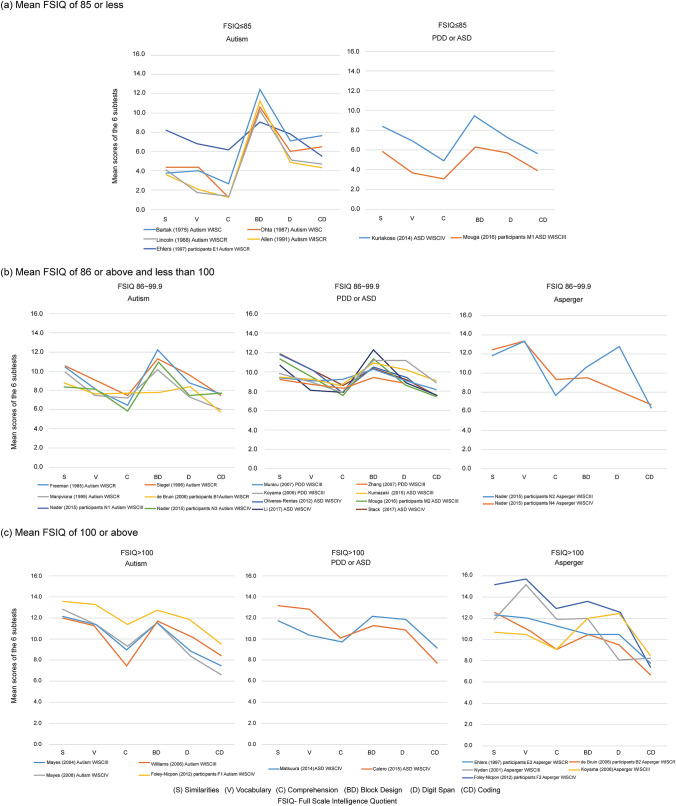
Comparison of six subtest profiles by diagnosis by intellectual level among three groups. **a** A comparison of six subtest profiles by diagnosis for those with a mean FSIQ of ≤ 85. **b** A comparison by diagnoses for those with a mean FSIQ of ≥ 86 and < 100. **c** A comparison by diagnosis for those with a mean FSIQ of ≥ 100. For those, a mean FSIQ of ≤ 85, no cases corresponded to an Asperger’s diagnosis. The order of subtests in figures for this study followed the order in the present WISC-IV manual. *ASD* Autism Spectrum Disorder, *PDD* Pervasive Developmental Disorder, *WISC* Wechsler Intelligence Scales for Children

Of the six groups with a mean FSIQ between 86–100 with autism diagnoses, except for de Bruin et al. (2006), the groups did not display extremely low verbal levels; however, they indicated troughs for Comprehension and peaks for Block Design, demonstrating islets of ability profiles. Conversely, while some diagnoses relating to PDD or ASD and Asperger’s confirm islets of ability profiles, they differed from the typical profile by having either Coding levels that were lower than Comprehension or their scores for Similarities, Vocabulary, or Digit Span levels were higher than Block Design rather than exhibiting the typical trough for Comprehension and peak for Block Design.

Among the groups with a mean FSIQ of ≥ 100, low Comprehension levels and high Block Design levels were observed among the four groups having autism diagnoses (in Foley-Nicpon et al. (2012) participants with autism diagnoses (F1); Mayes and Calhoun (2004, 2008); Williams et al. (2006b). However, the levels for Similarities and Vocabulary were higher than for Block Design, while Coding was lower than Comprehension, thereby resulting in an overall right-descending profile. Matsuura et al. (2014) and Calero et al. (2015) included PDD or ASD diagnoses showed similar right-descending profiles where Comprehension was lowest among the verbal tests and Coding was lowest in the overall test scores. Among the Asperger’s diagnoses group, high Similarities, Vocabulary, and Comprehension, together with low Coding test score levels were observed, resulting in overall right-descending profiles.

### High Block Design and Low Coding

As seen in Table 2 and Fig. 1, the mean scores for Block Design levels were 8 or above for all 32 groups, excluding de Bruin et al. (2006) participants with autism diagnoses (B1) and Mouga et al. (2016) participants with low IQ scores (M1). In addition, most scored at a consistently high level with a mean score higher than the average level of 10–12. Although the prominence of high Block Design was unnoticeable because of the high language levels among the recently reported high-IQ cases, this review reaffirmed that Block Design remained consistently high.

Participants with right-descending profile and those with classic islets of ability showed Coding scores that were not high (6–8) in either group. Also, the low Coding pattern has consistently been observed after the number of participants with high intellectual levels began to increase in published studies; thus, resulting in WISC patterns differing from the classic islets of ability.

Furthermore, this trend of high Block Design and low Coding was seen across all groups, irrespective of intellectual levels or diagnosis. Therefore, this trend can be confirmed as a cognitive characteristic that exists widely among all ASD groups.

## Discussion

This paper reviewed reports from 1970 to 2017 related to the ASD classic profile “islets of ability” (Lockyer and Rutter 1970). After examining the six subtest profiles based on intellectual levels and diagnosis, it was observed that the classic islets of ability profile was present in the autism diagnosis group with a mean FSIQ of < 100 and the PDD or ASD diagnosis group with a mean FSIQ of ≤ 85. On the other hand, for those groups with a mean FSIQ of ≥ 100 regardless of the diagnoses, the PDD or ASD diagnosis groups with a mean FSIQ of ≥ 86, and all Asperger’s diagnosis groups regardless of intellectual levels demonstrated right-descending profiles with high Similarities, Vocabulary, and Block Design together with low Coding rather than the classic islets of ability profile. In other words, the WISC profile of ASD individuals tended to demonstrate classic islets of ability profiles for those with low intellectual levels, whereas those with higher intellectual levels displayed right-descending profiles were.

In recent years, the prevalence of high-IQ score ASD has increased, and our results suggest that the WISC profile of children with ASD has transformed from the time when Kanner (1943) and Rutter (1978) proposed the concept of autism that centered on cases accompanying intellectual disability. Thus, answers to questions like “can any cognitive characteristics of ASD be captured by WISC?” and “can they exist perpetually without change?” are addressed in this study by demonstrating that high Block Design levels and low Coding levels continued to exist at the same levels, regardless of intellectual level, diagnostic name, and time-based changes.

This Block Design peak has been previously explained by Happe (1994) and Shah and Frith (1983, 1993) using the central coherence theory. Examples of related behaviors to the Block Design peak in ASD children include incredible dexterity in mold-fitting puzzles and in completing jigsaw puzzles. Today, ASD children with high intellectual ability are also patients of clinical practice, and the islets of ability on the WISC profile became less noticeable due to higher scores centered on the verbal test. However, Block Design scores remain high. In other words, the recent high scores of Block Design among children with ASD also indicate that good local information processing by weak central coherence is represented, despite being different from the previous profile on WISC.

Meanwhile, the low Coding in ASD were often discussed from the viewpoint of problems with motor coordination (Mayes and Calhoun, 2003; Szatmari et al. 1990). Hedvall et al. (2013) focused on the processing speed of children with ASD, and stated that Processing Speed subtests challenge the child’s capacity to work independently according to a given template and that they require graphomotor speed, accuracy, and mental flexibility/set shifting capacity to sustain attention to task, pointing out the effect of cognitive flexibility (Hedvall et al. 2013). In fact, observations of children with ASD during Coding tests indicate that even if there is no problem with the manual dexterity, the number of tasks that can be tackled is limited due to the difficulty of cognitive flexibility that shifts their attention toward next tasks. Such cases are not uncommon. Because the Coding results of children with ASD are associated with two problems, the problem with visuomotor coordination and the problem with cognitive flexibility, these scores can be considered consistently low.

Thus, if the evaluation scores of verbal tests (e.g., Similarities, Vocabulary, and Comprehension) are low together with consistently low Coding levels and contrastingly consistent high Block Design levels, these high Block Design levels would result in a prominent peak, thereby appearing as a classic islets of ability profile. In cases of individuals with high-IQ scores and high verbal scores, these verbal scores also create peaks that make the high Block Design less prominent, which might underline their right-descending profile.

Although the number of children with ASD with no islets of ability on WISC has increased in recent years, underlying problems of weak central coherence, cognitive flexibility and visuomotor coordination are still present. In fact, central coherence is associated with language and social development of ASD (Engel and Ehri 2020; Pellicano 2010), and poor cognitive flexibility was indicated to be related to the rigid and concrete bound behavior, occasionally transforming into perseverations of ASD (Lopez et al. 2005; Ozonoff and Jensen 1999). For example, cases in which children demonstrate difficulty seeing the whole picture because they concentrate too much on details, or cases in which they experience difficulty changing their perspectives once they presume are also observed frequently among children with ASD. Therefore, it may be of significant benefit for assessments and interventions to focus on weak central coherence and cognitive inflexibility to support such behaviors in clinical practice with children with ASD.

In this review, we demonstrated that the patterns of subtest performance of children with ASD are consistent with weak central coherence and limited cognitive flexibility. However, this observation cannot be interpreted as strong support for those hypotheses, because the individual studies included in the review did not disclose direct evidence that the subtest performance patterns are causally related to weak central coherence or cognitive inflexibility. Having said that, we still believe what we demonstrated constitutes beneficial reference material for clinicians to utilize when interpreting the WISC performances.

Today, ASD is known to be a clinical entity that combines multiple heterogeneous diseases. Based on the present review, when these diseases were combined into one, as ASD, the high Block Design performance and the low Coding performance on the WISC test were recognized as the coexisting neurocognitive endophenotypes. From this perspective, clarifying the path of how the neurocognitive endophenotypes will develop into the clinical phenotype of ASD should be an area of focus for future research (Viding and Blakemore 2007). Our proposition as a potential mean of clarifying this path is to examine the relationships between the clinically evaluated scores representing mentalizing, central coherence and cognitive flexibility, and the scores of Block Design as well as Coding among different types of ASD, ideally at several age points along the developmental course. Such examinations would reveal the mechanism of the endophenotypes’ development into the clinical phenotypes of ASD.

One of the limitations of this study was using only the six traditional subtests as subjects, thereby negating the examination of the other subtests. Each time WISC is revised, new subtests are also incorporated. In the future, the accumulation of knowledge about other subtests and newly adopted tests will therefore be required.
